# The Optical Effective Attenuation Coefficient as an Informative Measure of Brain Health in Aging

**DOI:** 10.3390/photonics6030079

**Published:** 2019-07-12

**Authors:** Antonio M. Chiarelli, Kathy A. Low, Edward L. Maclin, Mark A. Fletcher, Tania S. Kong, Benjamin Zimmerman, Chin Hong Tan, Bradley P. Sutton, Monica Fabiani, Gabriele Gratton

**Affiliations:** 1 Beckman Institute for Advanced Science and Technology, University of Illinois at Urbana-Champaign, Urbana, IL 61801, USA; 2 Department of Neuroscience, Imaging and Clinical Sciences, University G. D’Annunzio of Chieti-Pescara, 66100 Chieti, Italy; 3 Psychology Department, University of Illinois at Urbana-Champaign, Champaign, IL 61820, USA; 4 Division of Psychology, Nanyang Technological University, Singapore 639818, Singapore; 5 Department of Pharmacology, National University of Singapore, Singapore 117600, Singapore; 6 Department of Bioengineering, University of Illinois at Urbana-Champaign, Urbana, IL 61801, USA

**Keywords:** diffuse optical imaging (DOI), effective attenuation coefficient (EAC), aging, cortical thinning, FreeSurfer

## Abstract

Aging is accompanied by widespread changes in brain tissue. Here, we hypothesized that head tissue opacity to near-infrared light provides information about the health status of the brain’s cortical mantle. In diffusive media such as the head, opacity is quantified through the Effective Attenuation Coefficient (EAC), which is proportional to the geometric mean of the absorption and reduced scattering coefficients. EAC is estimated by the slope of the relationship between source–detector distance and the logarithm of the amount of light reaching the detector (optical density). We obtained EAC maps across the head in 47 adults (age range 18–75 years), using a high-density dual-wavelength optical system. We correlated regional and global EAC measures with demographic, neuropsychological, structural and functional brain data. Results indicated that EAC values averaged across wavelengths were strongly associated with age-related changes in cortical thickness, as well as functional and neuropsychological measures. This is likely because the EAC largely depends on the thickness of the sub-arachnoid cerebrospinal fluid layer, which increases with cortical atrophy. In addition, differences in EAC values between wavelengths were correlated with tissue oxygenation and cardiorespiratory fitness, indicating that information about cortical health can be derived non-invasively by quantifying the EAC.

## 1. Introduction

It is commonly accepted that, even in the absence of mild-cognitive impairment or Alzheimer’s Disease, aging is often associated with some degree of cortical atrophy, as manifested by cortical thinning [1-4], and that this atrophy can be accompanied by decrements in brain and cognitive function [5-8]. Measures of cortical thinning are typically obtained with structural magnetic resonance imaging (sMRI), which provides detailed images of the cortical layers that can be analyzed using semi-automated software (e.g., FreeSurfer© [9]). However, sMRI is an expensive tool to be used for screening normally aging adults, precluding its routine applicability in small clinics or for ambulatory use.

Diffuse optical imaging (DOI) was introduced a few decades ago as an alternative brain imaging modality, for its potential portability and relatively low cost. Initially, DOI was proposed as a tool for studying functional changes associated with variations in oxy- and deoxy-hemoglobin concentration (functional near-infrared spectroscopy, fNIRS) [10,11] and/or in neural activity (fast optical signal and event-related optical signal, EROS [12]). Although promising, these measures have remained less popular than measures based on functional magnetic-resonance imaging (fMRI) or electrophysiology (electroencephalography, EEG and event related brain potentials, ERPs). In part, this is because of the relatively low signal-to-noise ratio (SNR) of the approach.

We recently introduced another DOI-based measure, pulse-DOT [13], which, through a diffuse optical tomographic (DOT) approach, provides information about cerebrovascular status by estimating parameters of the pulse wave propagating through the brain’s arteries. Arterial stiffening (i.e., arteriosclerosis) has a profound influence on the health of cortical tissue. In fact, pulse-DOT estimates have been shown to be associated with aging, cardiorespiratory fitness, cerebrovascular reactivity and cognitive performance in adults [13-15], as well as with brain hemorrhaging in preterm infants [16].

Both fNIRS and pulse-DOT, which estimate the relative changes in the hemodynamic signal that occur with brain activation (fNIRS) and the cardiac cycle (pulse-DOT), can be obtained by measuring light attenuation with simple and cheap continuous-wave (CW) instruments. However, measures taken with CW instruments cannot provide separate quantitative estimates of the *absolute* optical properties of tissue (scattering and absorption). Absolute measures of these properties typically require time–domain (TD) or frequency–domain (FD instruments, to estimate photon pathlengths within the tissue and to uncouple absorption from scattering [17,18].

TD- and FD-based measures are technically complex and expensive, making absolute DOI a niche technology. However, absolute measures could in principle be very useful for assessing brain health, because NIR light is sensitive to the structural properties (head layers) and oxygenation levels of brain tissue. Recently, we have shown that CW recordings can in fact be used to derive an important baseline optical quantity, the effective attenuation coefficient (EAC) [19], which is proportional to the geometric mean of the absorption and reduced scattering coefficients [20]. Although EAC estimates do not uncouple absorption from scattering, they provide a summary measure of these two important optical parameters. The EAC can be estimated by measuring light decay as a function of source–detector distance [20]. At a sufficient distance from a source (~10 mm), light behaves as a purely diffusive field, decaying in an exponential manner and at an approximately constant rate. This means that the logarithm of light intensity, technically defined as Optical Density (OD), decays linearly with distance. The associated slope of light decay with distance is the EAC. Thus, the EAC provides information about how “opaque” to NIR light a diffusive medium is, with the EAC being generated by the interaction between structure inhomogeneity at the microscopic level (leading to optical diffusion, or light scattering) and tissue chromophore concentration (leading to light absorption). Note that the EAC may vary as a function of light wavelength, which reflects both the diffusion and absorption properties of tissue.

Using both simulations and physical phantom models, our group demonstrated that the CW-approach for estimating the EAC is accurate, if high-density optical arrays with many overlapping optical channels are employed. Importantly, we also showed that the EAC can be measured over most of the scalp (when using a large field-of-view optical recording montage), and that low-resolution maps of the EAC can be obtained for different areas of the head. These maps can reveal the presence (and, to some extent, the location and shape) of large inhomogeneities within the explored volume, as well as basic optical properties of various regions of the head [20].

In this paper we hypothesized that the EAC measured with this approach could provide information about several different parameters related to brain health. First, we hypothesized that the EAC could provide information about cortical atrophy. OD measures can be used to estimate the (weighted) average EAC of superficial (i.e., within the first 20–30 mm from the scalp) layers of the head and brain. Of particular importance is the fact that the EAC is relatively similar for most head tissues investigated by DOI, with the exception of (a) the cerebrospinal fluid (CSF) contained in the sub-arachnoid space (which is largely clear, with very low absorption and scattering, and therefore low EAC); and (b) large blood vessels (veins and arteries) running on the surface of the brain (which have very high absorption because of their high hemoglobin concentration). Note, however, that whereas the CSF forms a continuous layer covering the entire brain, the large blood vessels are highly localized. Thus, the thickness of the CSF layer has a large effect on the average EAC measured across the whole head, while the effects of large blood vessels are only visible at specific locations. As a consequence, we would expect the average EAC to be particularly large when the CSF layer is thin, and particularly small when the CSF layer is thick. In humans, the thickness of the CSF layer is a strong indicator of cortical (and, more generally, brain) atrophy: as brain tissue shrinks with aging or disease, it is replaced by CSF. Therefore, the more atrophic the brain, the thicker the CSF layer, and, consequently, the lower the brain’s average EAC. Thus, the EAC should be strongly correlated with measures of cortical atrophy (e.g., cortical thinning measured with sMRI). That being the case, we could in principle infer the level of brain atrophy from the EAC recorded across the head (at least in relative terms).

To test this hypothesis, we used a high-density optical montage to derive maps of EAC across the whole scalp in a sample of healthy participants (N = 48) varying in age between 18 and 75 years. The sample was selected to have an equal number of participants and an equal number of males and females for each decade of age (four in each group), allowing us to study how the EAC varies as a function of age and sex. We also obtained, in the same sample, T1w MR images for each participant, from which several structural brain parameters were derived using FreeSurfer© [9], including cortical thickness, cortical volume, and white matter signal abnormalities (WMSA).

Cortical atrophy in aging is known to be associated with cognitive decline [21-23]. As such, a corollary of the hypothesis that the EAC could provide an estimate of cortical atrophy, is that variations in EAC should also be correlated with variations in cognitive function. Cognitive function was therefore evaluated in the same sample with a battery of neuropsychological tests, focusing on working memory, executive function, and fluid intelligence (functions known to be affected by age and/or structural integrity) [2,24,25].

A second feature of the EAC, which may be useful for characterizing brain health, is its wavelength dependency (i.e., spectral features). In fact, within the NIR range, small changes in light wavelength generally cause small changes in light scattering, but large changes in absorption due to the strong sensitivity of NIR light to the hemoglobin species. This feature can be exploited to estimate tissue oxygen saturation from multi-wavelength EAC estimates [26]. Brain tissue oxygenation is expected to correlate with other age-related parameters linked to health, such as cardiorespiratory fitness (CRF; estimated using the Jurca et al.’s approach) [27], heart rate variability (HRV), and cerebrovascular status (estimated using pulse-DOT) [14]. Therefore, in the same sample, we also evaluated the association between the EAC and these parameters.

## 2. Materials and Methods

### 2.1. Participants

Forty-eight healthy adults (25 females) between the ages of 18 and 78 years were recruited into the study. Participants were stratified in order to obtain a uniform age and sex distribution (i.e., approximately 4 men and 4 women for each decade of age). All but one of the participants were Caucasians, which is a limitation of the study. Although not tracked, hair colors and densities were heterogeneous. Data from all participants are included, regardless of differences in skin tone or hair color and density. All participants were right-handed (as assessed by the Edinburgh Handedness Inventory) [28], reported no history of neurological or psychiatric disorders, and had no signs of dementia (as assessed by the modified Mini-Mental Status examination) [29] or depression (as assessed by the Beck’s Depression Inventory) [30]. The study was approved by the Institutional Review Board of the University of Illinois, and participants signed informed consent. Please note that the participants in this study are from the same sample included in [14,15], but that none of the analyses involving the EAC presented in this paper have been published before.

#### 2.1.1. Estimation of Cardiorespiratory Fitness

CRF estimates were obtained for each participant using the regression model proposed by Jurca and colleagues and others [27,31,32]. This model is based on weighted data that include gender, age, body mass index (BMI), resting heart rate, and a physical activity score. The CRF score is expressed in metabolic equivalents (METs), which are defined as the amount of oxygen consumed while sitting at rest [33].

#### 2.1.2. Neuropsychological Testing

The following neuropsychological tests were administered to all participants: Forward and Backward digit span, to measure working memory [24]; the Trail Making Tests A and B [34], to measure processing speed and working memory; the Controlled Oral Word Association sub-test of the Multilingual Aphasia Examination (a measure of verbal fluency using the letters CFL [35]; the Operation-Span task [36] to assess working memory capacity under load; Raven’s progressive matrices [37] and the Kaufmann Brief Intelligence Test Second Edition (K-BIT2) [38] to assess, respectively, cognitive flexibility and IQ; the vocabulary sub-test of the Shipley-Institute of Living Scale [39].

Note that several of these neuropsychological measures are highly correlated to each other, and therefore, in part, redundant. For this reason, and to reduce the number of comparisons, we created composite scores from individual tests to assess participants’ performance in specific cognitive domains, using an approach similar to Chan and colleagues [40]. Specifically, the neuropsychological tests were sorted into two different domains, which we labeled “performance” and “verbal”, respectively. The “performance” domain included the following scales: Raven’s matrices, forward and backward digit span, O-Span, trail A, trail B, and trail B-A. The verbal domain included Shipley’s vocabulary and verbal fluency. For each scale, the individual scores obtained by each participant were standardized, and, when needed, changed so that positive values always indicated a “better-than-average” score and negative values indicated a “worse-than-average” score. Then, for each participant the standardized values for the tests in each domain were averaged together (effectively giving each of them equal weight). These yielded two scores for each individual, a “performance” score and a “verbal” score. These scores should be interpreted as “summary” scores describing the major cognitive abilities of different individuals.

### 2.2. Collection and Processing of sMRI Data

Structural MRI data were collected for each participant using a 3T Siemens Trio full body scanner. A high resolution, 3D MPRAGE protocol was used, with a flip angle = 9°, TE = 2.32 ms, TR = 1900 ms, and inversion time = 900 ms. Slices were obtained in the sagittal plane (192 slices, 0.9 mm slice thickness, voxel size 0.9 × 0.9 × 0.9 mm) having matrix dimensions of 192 × 256 × 256 (in-plane interpolated at acquisition to 192 × 512 × 512) and field of view of 172.8 × 230 × 230 mm.

**Figure 1.**
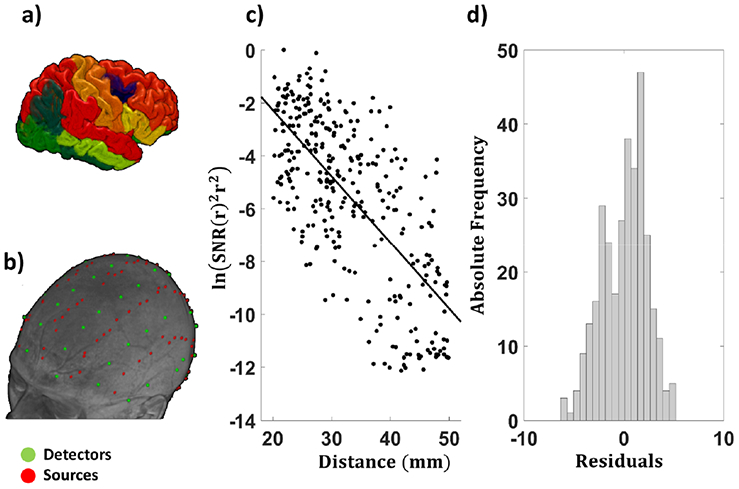
(**a**) Example of the regional cortical segmentation obtained with Freesurfer© 5.3 on the sMRI of the same participant. Different regions are color-coded; (**b**) High-density, large field-of-view, optical montage used for recordings, rendered onto the sMRI of a representative participant; (**c**) Left-hand side of Equation 1 (a value related to the log transform of light intensity) displayed for each channel as a function of source–detector distance for a representative subject. Only channels with acceptable (20–50 mm) source–detector distances for the 830 nm wavelength are shown; (**d**) Distribution of the residuals of the linear fit between inter-optode distance and the left-hand side of Equation (1).

FreeSurfer© 5.3 [9,41-45] was used to extract cortical information for each participant. All output data from FreeSurfer© were visually inspected by two trained reviewers, and errors were corrected according to standard methods recommended by the Martinos Center for Biomedical Imaging (for additional information, see https://surfer.nmr.mgh.harvard.edu/fswiki/FsTutorial/TroubleshootingData). Average cortical volume and thickness were computed for each individual. To account for differences in head size, cortical metrics were normalized by the estimated intracranial volume provided by FreeSurfer© 5.3 using the method first described by Jack and colleagues [46]. Figure 1a shows an example of regional cortical segmentation performed by Freesurfer© 5.3, with color-coding identifying different regions. Average cortical thickness and volume estimates were provided by averaging the 50 superficial cortical regions that were sufficiently close to the scalp to be relevant for optical recordings [14].

FreeSurfer© can also be used to quantify WMSA (i.e., small areas of demyelination that appear as hyperintensities in T2w MR images, and as hypointensities in T1w MR images [47,48]). WMSA are considered to be a sign of cerebral small vessel disease, a degenerative condition typically associated with cognitive decline, and may be a precursor of vascular and other forms of dementia [49]. In the current study, the estimates of WMSA based on T1w images were normalized by the subjects’ intracranial volumes before further analysis.

### 2.3. Optical Data Collection

Optical data were acquired with a multi-channel frequency–domain oximeter (ISS Imagent™, Champaign, IL, USA) equipped with 128 laser diodes (64 emitting light at 690 nm and 64 at 830 nm) and 24 photo-multiplier tubes (PMTs). NIR light was carried from the laser diodes to the scalp using single optic source fibers (0.4 mm core) and from the scalp back to the PMTs using detector fiber bundles (3 mm diameter). The fibers were held in place using custom-built soft helmets, the sizes of which differed as a function of head circumference. Time-division multiplexing was employed so that each detector picked up light from 16 different sources at different times within a multiplexing cycle. The sampling rate was 39.0625 Hz. Although the instrument also recorded phase-delay data, only intensity data were used in the current study, de facto using it as CW technology.

Source and detector locations (defined as the points of contact of the fibers with the head) were digitized with a Polhemus FastTrak 3D digitizer (Colchester, VT, USA; accuracy: 0.8 mm) using a recording stylus and three head-mounted receivers, which allowed for small movements of the head in between measurements [50]. During recording, participants performed a resting-state paradigm [51]. Two 6-min blocks were recorded for each of four consecutive periods in which different optical recording montages were used, for a total recording session of approximately 3 h (including the time required for setting up new optical montages). The helmet was never removed across the whole optical recording session.

A total of 384 channels (192 at 830 nm and 192 at 690 nm) were acquired for each montage, with source–detector distances varying between 15 mm and 80 mm for a total of 1536 channels (768 per wavelength) covering most of the scalp surface. Variability in source–detector distances is critical for measuring the EAC. Figure 1b shows source and detector locations, rendered onto the structural MR image of a representative participant.

### 2.4. EAC Computation

EAC values were computed employing the algorithm reported by Chiarelli et al. [20], where this procedure was described in detail. Here we only report information essential for understanding the current study. The algorithm estimates the EAC based on the slope of the log SNR of the recorded signal as a function of source–detector distance. In fact, using the simplifying assumption that the head can be approximated by a semi-infinite, homogenous medium with zero boundary conditions [19], the continuous-wave SNR recorded at a distance *r* from a source SNR(*r*) is linked to the EAC (μ_eff_) through the following formula:

(1) $$\text{ln}(\text{SNR}(r{)}^{2}r^{2})=k-r\mu_{\text{eff}}$$

where *k* is a factor that depends on μ_eff_ but does not depend on distance and is affected by source power, detector efficiency, and fiber coupling, and μ_eff_ is the EAC defined as:

(2) $$\mu_{\text{eff}}=\sqrt{3\mu_{a}(\mu_{a}+\mu_{s}\prime )}\approx \sqrt{3\mu_{a}\mu_{s}\prime }$$

if we assume that $\mu_{a}\ll \mu_{s}^{\prime }$ (as it normally is in head tissue in the NIR range). Equation (1) is valid assuming:

(3) $$I_{\text{light}}\propto {\text{SNR}}^{2}$$

where I_light_ is the amount of light reaching the detector. Equation (3) holds if noise is mainly due to quantum/shot noise (which is true if the amount of light detected is not extremely low and the SNR is computed using frequencies much higher than the physiological signal spectral range, >10 Hz) [20].

The SNR of the signal is defined as:

(4) $${\text{SNR}}^{2}=\frac{1}{var(\frac{i(t)}{i_{\text{avg}}})}$$

where *var* is the variance operator, i(t) is the electrical signal recorded by the photodetector in the spectral range of interest and i_avg_ is the average electrical signal.

The SNR was estimated over time within the recording period. The optical CW intensity data (i.e., the average measures of the amount of light produced by a specific source and reaching a specific detector during a multiplexing interval) were normalized (by dividing them by the mean intensity across a block), movement corrected [52], and high-pass filtered above 10 Hz (Butterworth digital filter) to eliminate the effects of cerebrovascular phenomena (such as vasodilation or vasoconstriction) on OD variance. The approach computes the EAC for each channel (source–detector pair) using subsets of channels near (neighbors) to the channel being estimated (within the neighborhood radius distance) in a multi-distance configuration. The neighborhood radius is a free parameter of the algorithm and affects both image resolution and EAC accuracy in a trade-off fashion. For the present study, a neighborhood radius of 30 mm was employed for each wavelength, which ensured, for each subject, an average of 19 channels (SD = 2) to be employed for each channel’s EAC computation. Based on Monte Carlo simulations and phantom studies, the number of channels employed here should provide an error in EAC estimate of less than a few percent points [20], being able to dampen the noise introduced by heterogeneous optodes power, sensitivity and/or coupling with the scalp. In order to obtain reliable EAC estimates, for most analyses (unless otherwise specified) only channels with inter-optode distances between 20 and 50 mm were employed (where a linear log SNR decay with distance was clear for all the subjects, satisfying Equation (1)). Short source–detector distances were excluded in order to respect the assumption of linearity in log light intensity drop with distance (this assumption does not hold for source–detector distances below 20 mm and for a large range of optical properties, even in homogeneous media). Long source–detector distances were excluded because of the loss of linearity in log SNR decrease with distance (SNR becomes flat). The effect of loss in linearity at long source–detector distances is a strong and clear phenomenon that is clearly visible in the data [20]. Therefore, the same source–detector distance range was used for all individuals. On average, we used 742 channels (SD = 64) (371 channels per wavelength, SD = 32) per subject for a percentage of employed channels with respect to the total channels acquired (1536) of 48%.

Figure 1c shows the log SNR of each channel as a function of source–detector distance for one representative subject, together with the distribution of the residuals of the linear fit (Figure 1d). Only channels within the 20–50 mm distance range and using light at 830 nm are displayed. The clear Gaussian distribution of the residuals corroborates the linearity assumption for this range of source–detector distances.

In order to create average maps of the EAC and to compare maps across participants, each channel’s midpoint was warped into a circular 2-dimensional template commonly employed for depicting topographic information from electroencephalographic data [53] and EAC maps were estimated in this 2-dimensional template space through interpolation. This procedure accounted for inter-subject variability in head size and allowed for visualization of the average EAC maps. Note that this approach, which relies on a flattening projection of a 3-dimensional space, slightly distorts ventral compared to dorsal locations.

### 2.5. Examination of the EAC as a Function of Source–Detector Distance

When computing the average EAC estimates across the entire head, a large neighborhood radius (>>head radius) was used in order to include all acceptable channels (on average 371 channels per subject, SD = 32, for each wavelength). As mentioned in the introduction, if overall age differences in EAC were found, it is important to determine which brain layer is especially responsible, which requires depth estimations. The average EAC estimates described above are based on a broad range of source–detector distances, and therefore are unable to provide this level of detailed information. Therefore, we also computed separate EAC estimates for several ranges of source–detector distances (4 groups, 10 mm intervals; 15–25 mm, 25–35 mm, 35–45 mm, 45–55 mm). These data provide an indirect, approximate indication of the depth (which is monotonically related to the average source–detector distance used for the analysis, with depth values typically somewhat smaller than half of the source–detector distance [54]) at which the major changes in EAC occurred across subjects.

### 2.6. Computation of the Tissue Oxygenation Index

We were also interested in the ability of EAC estimates to provide information about an individual’s brain tissue oxygenation, a parameter of significant clinical importance. The average Tissue Oxygenation Index (TOI), which is equivalent to tissue oxygen saturation, was computed using the EACs at the two wavelengths with an algorithm reported by Suzuki and colleagues [26]. Tissue oxygenation level can be assessed with the TOI when spectral information regarding the EACs is available and spectral dependence of the reduced scattering coefficient is assumed. In general, the TOI is a function of the ratio of the EACs measured at the two wavelengths.

### 2.7. Computation of the Cerebral Arterial Pulse Relaxation Function Obtained with Pulse-DOT

The pulse relaxation function (PReFx) [13-15] is a measure of the shape of the pulse during the interval between a peak systole and a peak diastole. It describes the way in which arteries return to their original size after dilating to accommodate the blood bolus generated by a heart pulsation. To the extent that this curve is decelerated, the artery can be considered to be elastic; acceleration of this function is a sign of arterial stiffness [55].

Average pulses, locked to the peak of the electrocardiogram (EKG) R-wave, were computed for each recording channel. Channels at 830 nm were used for tomographic reconstruction of the pulse because of their higher SNR compared to those at 690 nm. In order to generate a 3D reconstruction of the pulse waveform across the head, a model of light propagation (forward model) and an inverse procedure are required. The Finite Element Method (FEM) applied to the diffusion equation [56,57] was used to estimate the forward model. The FEM software NIRFAST [51,58] was used to model light propagation through heterogeneous head models and to compute Jacobian (sensitivity) matrices of light intensity reflecting absorption changes. An inverse procedure [59] was used to convert intensity changes on individual channels to absorption changes in voxel space. PReFx was computed as the normalized area (based on duration and amplitude), under the pulse during the systole-diastole interval, after subtracting a triangle describing a “linear” relaxation. PReFx was estimated for each voxel for which the light sensitivity (measured by the average Jacobian) was greater than 1/1000 (60 dB) of the maximum value. This allowed us to disregard voxels that were too deep to provide useful data (i.e., >30 mm from the scalp) as well as voxels that were not covered by the optical montage. In addition, only voxels within the cortex (as identified by FreeSurfer©) were considered. The average PreFx was computed by integrating PReFx values from all voxels within the cortex. For additional information about the computation of PReFx using a DOT approach, please refer to [14].

### 2.8. Heart Rate and Heart Rate Variability

Participants’ heart rate and HRV were estimated from the optical data. Pulse signals were averaged across all acceptable channels to obtain a single pulsating signal for the recording period. The duration of each inter-beat interval was calculated as the time lag between two consecutive diastolic peaks. This allowed us to compute the mean and standard deviation of the inter-beat intervals for each participant. The inverse of the mean value was used to compute heart rate (HR, translated into beats per minute). The second value was used as an estimate of HRV (in ms).

### 2.9. Post-Processing and Statistical Analyses

Three series of analyses were performed on these data. The first series was aimed at investigating the topographic properties of the EAC and their reliability across the 48 subjects. Establishing such characteristics is essential for using the EAC to assess individual differences in parameters related to brain health. The EAC maps of each subject at each wavelength, warped to the circular bi-dimensional (i.e., flat) representation described earlier, were averaged across subjects, and means and standard errors across subjects were estimated for each pixel. Similarities between the EAC maps at the two wavelengths were estimated through correlation analysis.

The second series of analyses focused on a cross-sectional investigation of the associations between the average EAC, age, and other indices of brain status. Specifically, correlations were computed between the overall EAC obtained at each wavelength for each subject (computed using all acceptable channels) and age, cortical volume and thickness and tissue oxygenation (TOI). Because of the strong age effect on all these variables, correlations after partialing out the effect of age were also evaluated. A Principal Component Analysis (PCA) [60] was also performed to assess the main dimensions underlying the relationship between the EAC values computed using the light of different wavelengths.

The third series of analyses focused on mediation, aimed at elucidating the extent to which health-related brain parameters (namely, cortical thickness and tissue oxygen saturation) mediate the relationship between age and the first and second EAC principal components, respectively. The Baron and Kenny’s approach [61] was used to perform the mediation analyses. This approach involves three steps. The first two are aimed at demonstrating the presence of a significant relationship between the independent variable (e.g., age) and the dependent variable (e.g., EAC component 1 and 2), as well as between the independent variable (e.g., age) and the proposed mediator (e.g., cortical thickness and tissue oxygen saturation). If these relationships are not significant, it is not possible to talk about mediation. The final step involves regressing the dependent variable on both the independent variable and the mediator through multiple regression. After this step, the unique effect of the independent variable when the mediator is included as a predictor is compared to the simple effect of the independent variable alone. If including both the mediator and independent variable into the regression equation eliminates the dependent variable’s association with the independent variable, then the remaining significant variable is said to fully mediate the effects of the other on the dependent variable; if instead the dependent variable’s association with the independent variable is significantly reduced but still present, the mediator is said to partially mediate the association; finally if the reduction in correlation is not significant (or the correlation even increases), no mediation is considered to be found. A Sobel test was conducted to assess the statistical significance of the mediations [61].

## 3. Results

The Section 3 is divided into two parts. The first part provides basic statistics and reliability information about the EAC data. It also includes a series of analyses aimed at demonstrating that the EAC value (averaged across locations) provides information related to (a) the average thickness of the CSF-layer (subarachnoid space) in a given individual (when the EAC values computed using light at 690 and 830 nm wavelengths are averaged together), and (b) the average oxygenation of brain tissue (when the difference or ratio between the EAC values computed using light at 690 and 830 nm wavelengths is considered). These two pieces of information are reflected, respectively, by the first and second eigen-solutions (or principal components) of the space identified by the two EAC measures.

The second part is aimed at demonstrating that the first EAC eigen-solution (EAC1) is in fact strongly associated with cortical thickness (and therefore also with cognitive performance), whereas the second EAC eigen-solution (EAC2) is associated with cardiorespiratory fitness, providing indirect validation for its interpretation as a measure of tissue oxygenation and health.

We did run multiple tests of the null hypotheses, and did not correct for multiple comparisons for the following reasons: (a) most of these tests were significant at *p* < 0.001—which would be reflected in significant results if we had applied a Bonferroni approach (the most conservative method available for such corrections) across all correlations computed; (b) in several cases, it is difficult to determine how independent the different tests of the null hypotheses are, since there is a possibility of correlated error (making the Bonferroni approach needlessly conservative); (c) many of the correlations reported were predicted a priori; and (d) all null hypotheses’ *p* values were based on non-directional tests, despite the fact that our hypotheses were directional as relating to aging effects. This is an inherently conservative approach.

### 3.1. Basic Statistics and Interpretation of the EAC

#### 3.1.1. EAC Maps Characteristics

**Figure 2.**
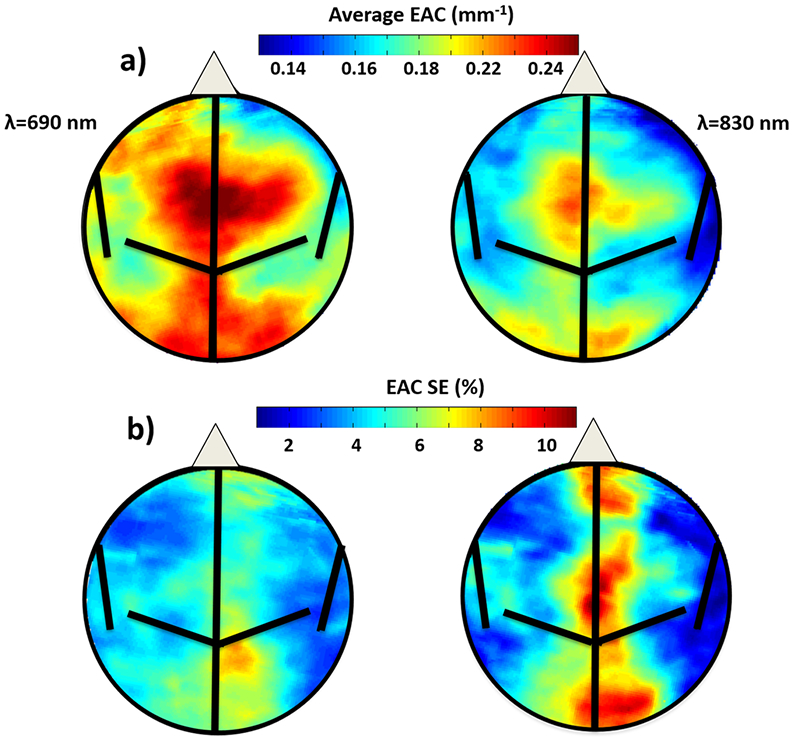
(**a**) Circle-warped grand average Effective Attenuation Coefficient (EAC) maps across all subjects at the two wavelengths (690 nm and 830 nm); (**b**) Maps of the EAC standard error (SE) of the mean (expressed as percentage of the average value for each pixel) for the two wavelengths (690 nm and 830 nm). Estimated central sulcus and lateral and longitudinal fissures of a 2D circle-warped brain are delineated in black.

Grand average EAC maps for each wavelength (690 and 830 nm) are reported in Figure 2a. The average EAC values across subjects ranged from 0.16 mm^−1^ to 0.22 mm^−1^ for the 690 nm map and from 0.14 mm^−1^ to 0.20 mm^−1^ for the 830 nm map (a variability of ~40%). The 690-nm map showed higher EAC values than the 830-nm map, indicating that the head is more transparent at longer NIR wavelengths.

A reliability test was conducted on the EAC maps obtained at each wavelength by randomly splitting the subjects into two groups (with age and gender evenly distributed between the groups). The pixel-wise correlations between the maps for the two groups were extremely high at both wavelengths (*r* = 0.994 for the 690 nm and r = 0.995 for the 830 nm). This indicates that group maps of the EAC are very reliable.

Clear similarities between the EAC maps across the two wavelengths were also evident, with dorsal and occipital areas showing higher EAC values than lateral and frontal regions (pixel-wise correlation between the maps obtained at each wavelength: *r* = 0.92). The standard errors of the mean (reported as percentages of the average value for each pixel) for the EAC maps are reported in Figure 2b. For most pixels the error was less than 10%, with pixels with larger EAC values having typically larger percent errors.

#### 3.1.2. Average EAC Characteristics

**Figure 3.**
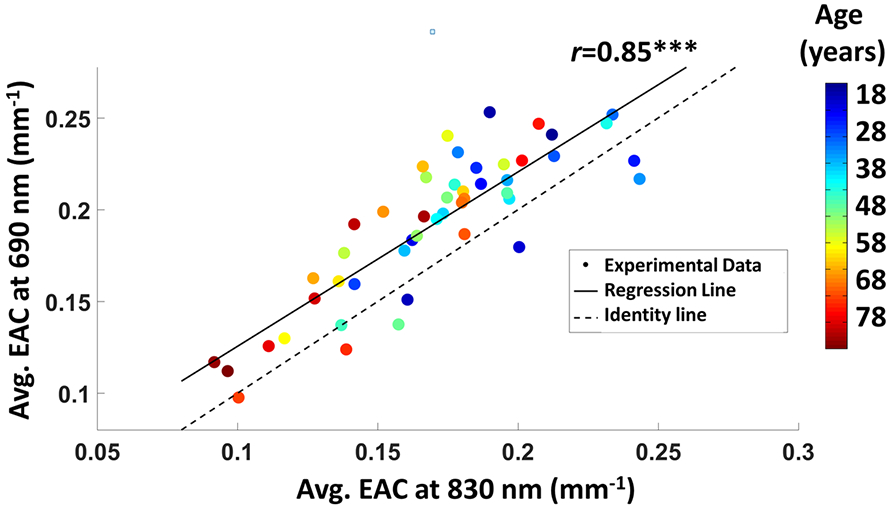
Average EAC values at 690 nm as a function of average EAC values at 830 nm. Each dot in the scatterplot is a participant, color-coded as a function of age (from blue to red in ascending age order). (*** *p* < 0.0001).

Figure 3 reports the EAC values measured in individual subjects and averaged across all locations at 690 nm, as a function of the EAC values at 830 nm. Each dot in the scatterplot is color-coded as a function of age (from blue to red in ascending age order). There was a significant correlation between the two wavelengths (*r*(46) = 0.85, *p* < 0.0001). EAC values ranged from 0.097 mm^−1^ to 0.253 mm^−1^ for 690 nm and from 0.092 mm^−1^ to 0.243 mm^−1^ for 830 nm, with average values (Mean ± SD) EAC_690_ = 0.192 ± 0.040 mm^−1^ (95% Confidence Interval, CI, 0.180–0.203 mm^−1^) and EAC_830_ = 0.170 ± 0.036 mm^−1^ (CI 0.160–0.180 mm^−1^). The comparison between the regression line (solid) and the identity line (dashed) in Figure 3 indicates that the EAC was larger at 690 nm compared to 830 nm (average difference ΔEAC = 0.022 mm^−1^, paired *t*-test *t*(47) = 7.19, *p* < 0.0001).

**Figure 4.**
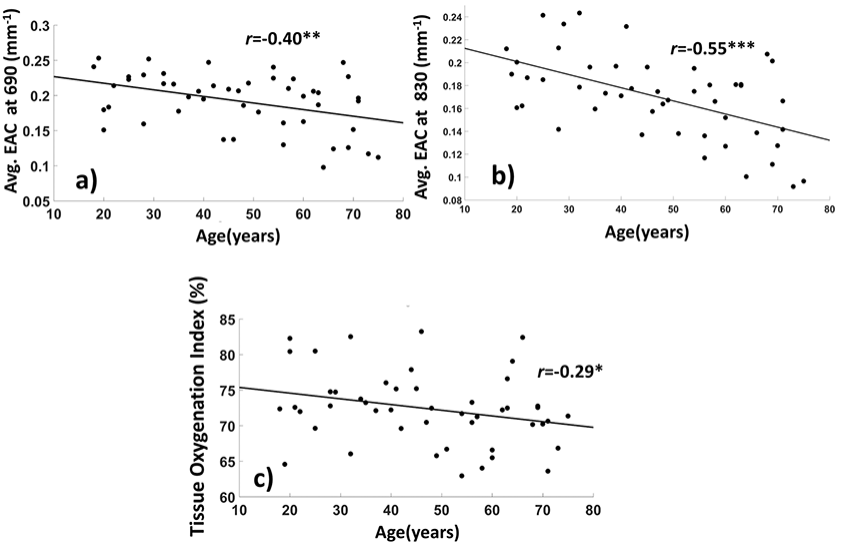
(**a**) Average EAC as a function of age for the 690 nm wavelength; (**b**) same for the 830 nm wavelength; (**c**) average Tissue Oxygenation Index (TOI) as a function of age. (* *p* < 0.05, ** *p* < 0.01, *** *p* < 0.001).

An age effect was apparent, with younger adults (blue color range) exhibiting higher EAC values than older adults (red color range). Figure 4a,b show the effect of age on the EAC for each of the two wavelengths (age vs. EAC: *r*_690nm_(46) = −0.40, *p* = 0.005; r_830_(46) = −0.55, *p* < 0.0001), with a rate of change (slope) in the EAC of ΔEAC_690nm_/year = −0.009 and ΔEAC_830nm_/year = −0.011. Because the longer wavelength is more sensitive to oxygenated hemoglobin, the greater age-related decrease in EAC for the 830 nm light could be attributed to decreasing levels of oxygenation with age. In fact, we also found a decrease in global TOI as a function of age (*r*(46) = −0.313, *p* = 0.030), shown in Figure 4c. We did not find any effect of sex on the age–EAC association.

**Figure 5.**
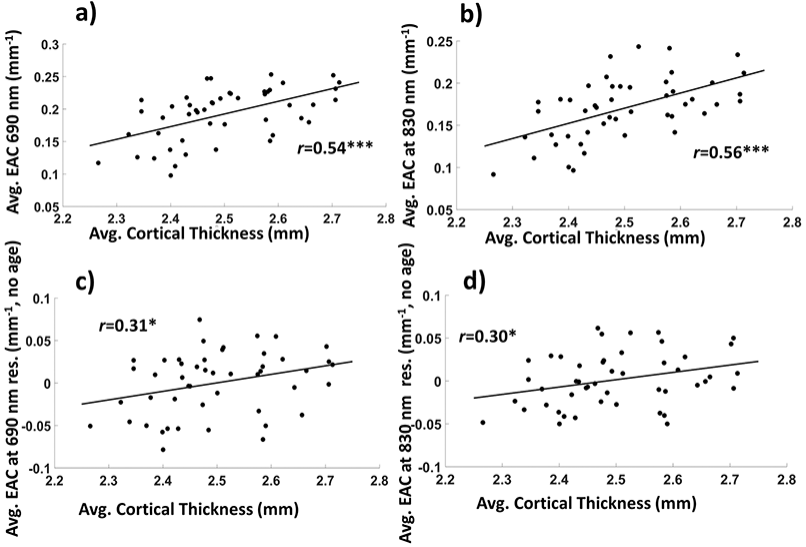
(**a**) Average EAC as a function of cortical thickness for the 690 nm wavelength; (**b**) same for the 830 nm wavelength; (**c**) Average EAC as a function of cortical thickness for the 690 nm wavelength, when age is partialed out (“no age”); (**b**) same for the 830 nm wavelength (* *p* < 0.05, ** *p* < 0.01, *** *p* < 0.001).

Figure 5a-d reports scatterplots of the EAC as a function of average cortical thickness as estimated with FreeSurfer©, with plots c and d reporting the same correlations as a and b after partialing out the effect of age. The EAC was positively correlated with cortical thickness for both wavelengths (*r*_690nm_(46) = 0.54, *p* < 0.0001; *r*_830nm_(46) = 0.56, *p* = <0.0001).

Interestingly, the EAC was also correlated, albeit to a lesser extent, with total cortical volume (cortical volume vs. EAC: *r*_690nm_(46) = 0.40, *p* = 0.005; r_830_(46) = 0.47, *p* < 0.0007). It should also be noted that, although the TOI, cortical thickness and cortical volume were all significantly correlated with age (age vs. cortical thickness: *r*(46) = −0.65, *p* < 0.0001; age vs. cortical volume: *r*(46) = −0.81, *p* < 0.0001. age vs. TOI: *r*(46) = −0.31, *p* < 0.032), no significant correlation was found between the TOI and brain volumetric estimates (TOI vs. cortical thickness: *r*(46) = 0.043, *p* = 0.77; TOI vs. cortical volume: *r*(46) = 0.15, *p* = 0.31). As expected, cortical thickness was correlated with cortical volume (*r*(46) = 0.78, *p* < 0.0001).

#### 3.1.3. Average EAC as a Function of Source–Detector Distance

**Figure 6.**
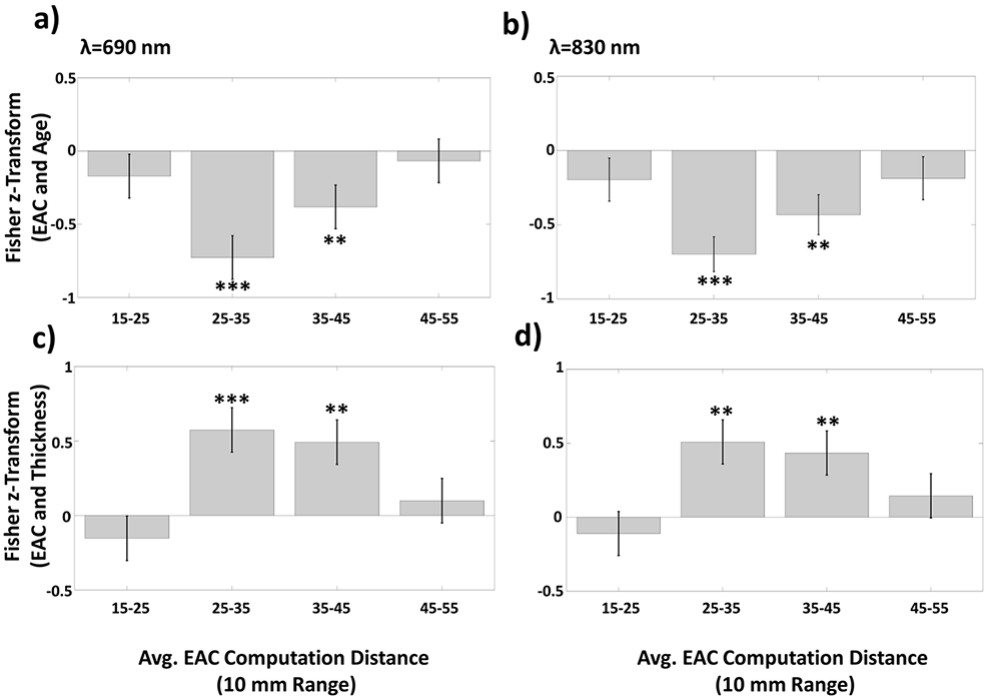
(**a**) Average Fisher transforms of correlation between EAC at the 690-nm wavelength and age (and related standard errors) as a function of inter-optode distance (4 groups: 15–25 mm, 25–35 mm, 35–45 mm, 45–55 mm); (**b**) Same as (**a**) for the 830-nm wavelength; (**c**) Average Fisher transforms of correlation between EAC at the 690-nm wavelength and cortical thickness (and related standard errors) as a function of inter-optode distance (4 groups: 15–25 mm, 25–35 mm, 35–45 mm, 45–55 mm); (**d**) Same as (**c**) for the 830-nm wavelength (* *p* < 0.05, ** *p* < 0.01, *** *p* < 0.001, *** *p* < 0. 0001).

To provide some initial indication of the depth within the head of the phenomena linking EAC to age and cortical thickness, we computed EAC separately for four source–detector distance ranges (considering that the average depth sensitivity of each channel is monotonically related to its source–detector distance) [54]. Note that, because of its effect on the depth of the area explored, source–detector distance can also be expected to influence the relative sensitivity of the measures to different types of tissue (scalp, skull, CSF, gray and white matter) [62]. The Fisher-z transform of the correlations between these EAC estimates with age and cortical thickness are shown in Figure 6. The standard error of the Fisher transform of the correlations was computed as (*N* – 3)^−1/2^ where N is sample size. Statistical analysis revealed significant correlations between EAC and age (Figure 6a,b) or thickness (Figure 6a,b) at intermediate inter-optode distance ranges, whereas no correlation was significant for the shortest and longest distance ranges. These data indicate that age- (and cortical-thickness-) related changes in EAC occur at source–detector distances between 25 and 45 mm, corresponding to a maximum penetration between 10 and 20 mm. With this range of penetration, it could be expected that the optical data would be relatively more sensitive to CSF and gray matter phenomena, and less sensitive to skin and skull phenomena [62].

#### 3.1.4. EAC Orthogonalization in Wavelength Space

The interpretation proposed earlier that the absolute value of EAC (averaged across all locations) might reflect anatomical factors related to brain atrophy, but that EAC may also provide information about physiological factors (such as tissue oxygenation) may be of theoretical and translational interest. However, it rests on the assumption that these two factors (i.e., latent variables) are independent from each other. This could be questionable, since both parameters are extracted from the same data. A way to determine whether they are in fact independent is to conduct a PCA (Eigen-decomposition) of the space defined by the EAC values obtained at each wavelength (see Figure 3) to determine whether independent component of variance, related to these two latent variables, can be extracted from the data.

**Figure 7.**
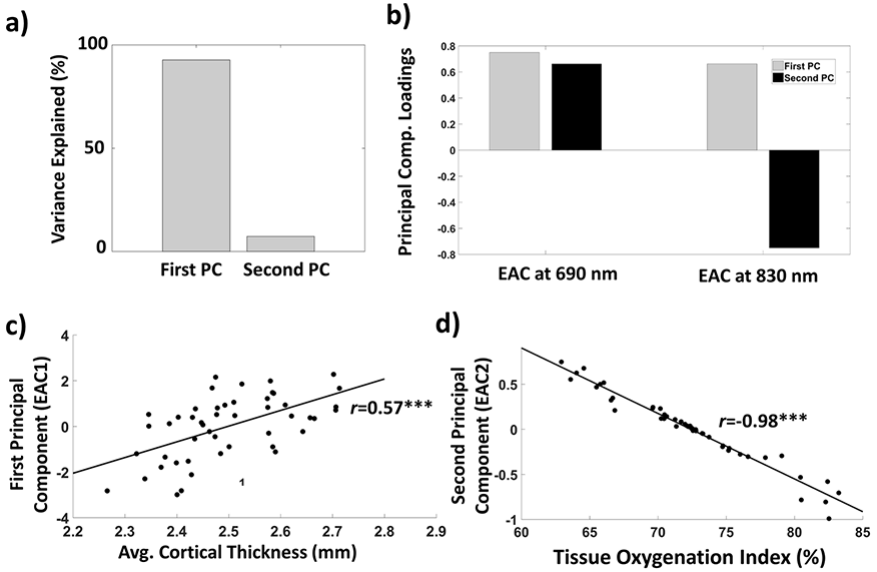
(**a**) Between-subject variance explained by the two principal components (PCs) of a PCA performed in the wavelengths’ space (690 nm and 830 nm); (**b**) Loadings for the first and second PC; (**c**) First PC as a function of average cortical thickness; (**d**) Second PC as a function of TOI. (*** *p* < 0.0001).

Specifically, we used PCA to decompose the variance in EAC between subjects at the two wavelengths into two orthogonal latent variables (eigen-solutions or principal components, PCs). The first PC (i.e., the axis of maximum variance) explained 92.76% of the variance, whereas the second orthogonal component explained the remaining 7.24% (Figure 7a). Although the loadings of the first PC for each wavelength were of approximately equal magnitude and sign (reflecting an equal contribution of each wavelength), the second PC had a positive loading for the 690 nm wavelength and a negative loading for the 830 nm wavelength (also approximately equal in magnitude but opposite in sign, Figure 7b). Therefore, we can at least approximately describe these two components as the average and the difference between the EAC measured at 690 and 830 nm, respectively. Both components were significantly correlated with age (First PC: *r*(46) = −0.49, *p* < 0.0001; Second PC: *r*(46) = 0.31, *p* < 0.032). Figure 7c reports a scatterplot depicting the relationship between cortical thickness and the first PC. Interestingly, the first PC was significantly correlated with cortical thickness (*r*(46) = 0.57, *p* < 0.0001) and cortical volume (*r*(46) = 0.45, *p* = 0.0013), with higher correlation values than each wavelength alone. In contrast, the second PC was not correlated with either cortical thickness (*r*(46) = −0.03, *p* = 0.84) or volume (*r*(46) = −0.12, *p* = 0.42). The opposite relationships were found when relating the PCs to TOI. The first PC did not significantly correlate with TOI (*r*(46) = −0.055, *p* = 0.71) whereas the second PC did (*r*(46) = −0.97, *p* < 0.0001; see Figure 7d). In summary, the first component appears to be related to anatomical sources of variance, whereas the second appears to be related to physiological parameters (such as tissue oxygenation), even though both components are correlated with age.

#### 3.1.5. Mediation Analyses

**Figure 8.**
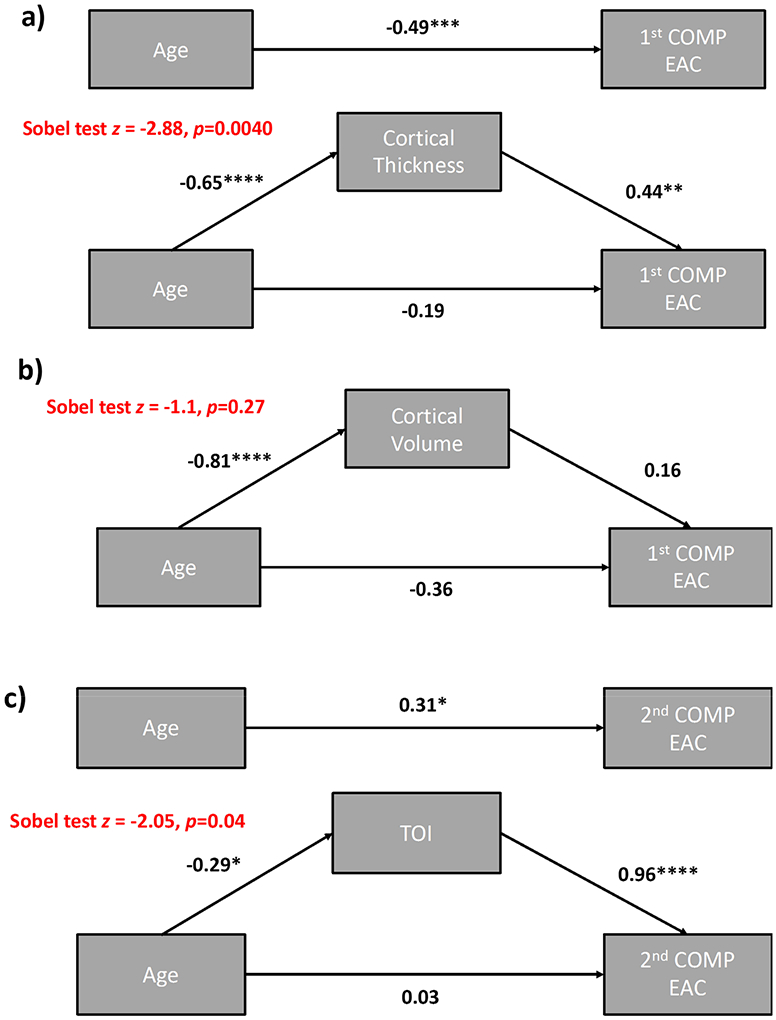
(**a**) Mediation analysis investigating the mediation effect of cortical thickness on the association between age and the first EAC component; (**b**) Mediation analysis investigating the mediation effect of cortical volume on the association between age and the first EAC component; (**c**) Mediation analysis investigating the mediation effect of TOI on the association between age and the second EAC component. *p < 0.05, ** *p* < 0.01, *** *p* < 0.001, **** *p* < 0.0001.

The first mediation analyses (Figure 8a) aimed to determine whether average cortical thickness exerts a mediating role on (i.e., statistically accounts for) the association between age and the first PC component of EAC. This analysis showed that cortical thickness fully mediates the age-related changes in the first PC component of EAC (age vs. first PC β_1_ = −0.19, *p* = 0.20, cortical thickness vs. first PC β_2_ = 0.44, *p* < 0.002, Sobel test *t* = −2.88, *p* < 0.006), supporting the hypothesis that the relationship between the first component of EAC (approximately the average EAC at 690 and 830 nm) and age mainly reflects the relationship between this parameter and cortical thickness (which is known to decline with age).

Similar mediation analyses were performed considering cortical volume instead of cortical thickness as the mediator between age and EAC at each wavelength and age and first PC of EAC (Figure 8b). These mediations were not significant (age vs. EAC_690_: β_1 690nm_ = −0.21, *p* = 0.157, cortical volume vs. EAC_690_: β_2 690nm_ = 0.22, *p* = 0.137, Sobel test *t* = −1.56, *p* = 0.126; age vs. EAC_830nm_: β_1 690nm_=−0.47, *p* = 0.0008, cortical volume vs. EAC_830nm_: β_2 830nm_ = 0.08, *p* = 0.593, Sobel test *t* = −0.57, *p* = 0.57; age vs. first PC: β_1_ = −0.36, *p* = 0.013, cortical volume vs. first PC: β_2_ = 0.16, *p* = 0.28., Sobel test *t* = −1.1, *p* = 0.28).

We also investigated whether tissue oxygenation (TOI) mediates age-related variability in the second PC of EAC (Figure 8c). This analysis indicated a full mediation effect (age vs. second PC: β_1_ = 0.03, *p* = 0.84, TOI vs. second PC: β_2_ = 0.44, *p* = 0.002, Sobel test *t* = −2.05, *p* = 0.046). It should be noted, however, that the second PC is derived from a linear decomposition of EACs in the wavelength space, whereas TOI is a non-linear function of EACs. Therefore, TOI and EAC are not really independent measurements, and this result should not be considered definitive.

### 3.2. Relationships between EAC Eigen-Solutions and Brain Anatomy, Cardiorespiratory Fitness, and Cognitive Performance

Correlations between the first and second EAC eigen-solutions (EAC1 and EAC2), age, and other demographics, physiological, brain, and cognitive variables are presented in Table 1. This table also includes correlations of these variables with EAC1 and EAC2 with age partialed out. In addition to confirming the relationships with age and cortical thickness (already discussed earlier for each of the wavelengths), EAC1 was significantly correlated with several other variables of interest, including white matter integrity (as indexed by white matter signal abnormalities, WMSA) and arterial elasticity (measured with the PReFx pulse-DOT parameter). Both of these correlations remained significant when age was partialed out. In addition, EAC1 was correlated with various psychological measures. Most of these cognitive measures we labeled “performance” reflect aspects of fluid intelligence, which is known to decline with age [63, 64]. In our study, this was reflected by the strong correlation between EAC1 and the Performance score. However, the strong collinearity between age, EAC1, and these functional/psychological measures makes it quite difficult to determine whether these associations are specific to EAC1 or merely reflect separate age effects on all these variables.

EAC2 had, in general, lower correlations than EAC1 with most of the variables. However, a significant correlation was found between EAC2 and HRV, an index related to cardiovascular function, and with verbal fluency. Both of these correlations were negative, reflecting the fact that higher EAC2 values were associated with lower levels of tissue oxygenation.

**Figure 9.**
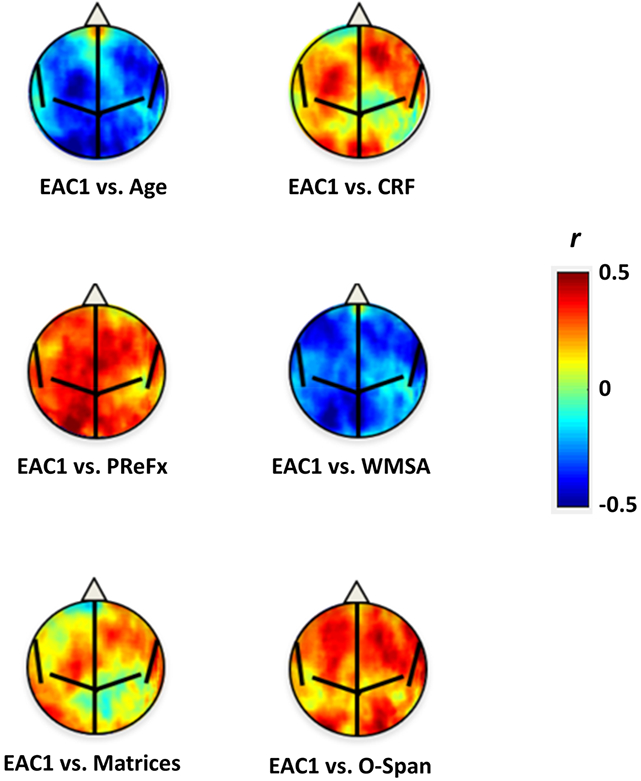
Circle-warped maps of the correlations between local measures of the first EAC component and some relevant variables in the study (Age, cardiorespiratory fitness (CRF), pulse relaxation function (PReFx), white matter signal abnormalities (WMSA), Matrices, O-Span). Estimated central sulcus and lateral and longitudinal fissures of a 2D circle-warped brain are delineated in black.

Figure 9 reports maps of the correlations between local EAC1 estimates (i.e., values of EAC1 computed for each pixel on the surface of the head) and several individual-difference variables of interest, including age, cardiorespiratory fitness (CRF), PReFx, WMSA, and two neuropsychological scales designed to indicate reasoning (matrixes) and working memory (O-Span) function. Several of these maps indicated that the strongest correlations could be observed when EAC1 was estimated from optical probes located over the frontal and parietal regions. This pattern was particularly evident for the O-Span measures.

## 4. Discussion

Accurate characterization of the optical properties of head and brain tissue is of great interest. This information is crucial for precise light path length estimation, which is needed for the accurate calculation of oxy- and deoxy hemoglobin concentration in fNIRS. Most importantly, NIR light propagation is affected by the anatomical and oxygenation properties of the structures being investigated and therefore could be sensitive to physiological and pathological conditions. Currently, no systematic mapping of the optical properties of head and brain tissue in a significant sample is present in the literature. This paucity of data is caused by technical difficulties in retrieving the photons’ time of flight information needed to separate absorption and reduced scattering coefficients throughout the diffusive structures of head.

We recently developed a procedure that maps the EAC over the head and cortical mantle using continuous-wave systems, and therefore does not require photons’ time-of-flight estimates [20]. The EAC (which is a combination of the absorption and reduced scattering coefficients) could be a useful tool for understanding how light propagates through the head, since it is the main parameter affecting light propagation in deep structures (for depths exceeding a few mm). Since measurement of the EAC is based on a multi-distance approach, application of the algorithm requires that a high-density optical array be used for recording the data. To generate extensive maps of EAC across the scalp (as in the current study), large recording arrays are necessary. However, if only local measures are of interest, a relatively small recording array, composed of as little as 16 channels, might be sufficient. Importantly, measurement of the EAC does not require additional manipulations, so that the EAC can be computed from the same uncalibrated data obtained in a standard fNIRS study employing CW technology (provided that an appropriate multi-distance montage is used).

In this paper we report a systematic mapping of the EAC in 48 healthy adults uniformly spread across a broad age range (18–78 years), with each decade equally encompassing both genders. We evaluated both the whole-head spatial characteristics of the EAC and its average cross-sectional correlations with age and brain anatomical (cortical thickness and volume, based on sMRI segmentations, and WMSA) and oxygenation parameters, as well as its correlations with measures of arterial stiffness, heart rate variability, cardiorespiratory fitness, and cognitive function. Our recording was based on large number of channels (1536), half using 690 nm light and the other half using 830 nm light. The spatial coverage of the two wavelengths was essentially identical, since the optic fibers carrying light of the two wavelengths were paired.

The EAC estimates retrieved with this method were consistent with previous estimates of the absolute absorption and reduced scattering properties of the human head obtained in vivo [65,66]. In particular, the results presented here were comparable with the most recent study on this topic using TD technology by Giacalone et al. [66]. In fact, although Giacalone and colleagues acquired TD data on only 12 pre-defined locations of the head according to the 10-10 international system (position F3-F5, C3-C1, P3-P5 for the left hemisphere and C4-C2, F4-F6, P4-P6 for the right hemisphere), the number of participants and age range were similar to our study. Their subjects’ average global values for absorption and reduced scattering coefficient were: μ_a_ = 0.0144 ± 0.0043 mm^−1^ (CI 95%, 0.0140–0.0148 mm^−1^) at 690 nm, $\mu_{\;s}^{\prime }$ = 0.830 ± 0.180 mm^−1^ (CI 95%, 0.810–0.850 mm^−1^) at 690 nm, μ_a_ = 0.0137 ± 0.0032 mm^−1^ (CI 95%, 0.0134–0.0140 mm^−1^) at 830 nm, $\mu_{\;s}^{\prime }$ = 0.720 ± 0.160 mm^−1^ (CI 95%, 0.700–0.730 mm^−1^) at 830 nm. These results corresponded to average EACs equal to: EAC_690nm_ = 0.189 ± 0.045 mm^−1^ (CI 95%, 0.184–0.194 mm^−1^) and EAC_830nm_ = 0.171 ± 0.036 mm^−1^ (CI 95%, 0.167–0.175 mm^−1^). These results are well within the confidence intervals (i.e., with differences of less than 2%) of the findings of the current study: EAC_690_ = 0.192 ± 0.040 mm^−1^ (Confidence Interval, CI 95%, 0.180–0.203 mm^−1^) and EAC_830_ = 0.170 ± 0.036 mm^−1^ (CI 95%, 0.160–0.180 mm^−1^).

Our data also indicate that the EAC varied regionally depending on where on the head the data were recorded from. For both wavelengths dorsal and occipital areas of the head showed higher EAC estimates than lateral or frontal regions (Figure 2a). In fact, these topographic differences are not small, with EAC varying by a factor of ~40% depending on the location of the head where it is measured. When comparing these results with the recent work by Giacalone and colleagues, they also found regional variability in absolute optical properties, with central regions (C3, C1; locations close to dorsal areas) having higher absorption and reduced scattering coefficients compared to frontal or parietal regions. These absorption and reduced scattering coefficients corresponded to highest EAC values in central regions: EAC_690_ = 0.195 ± 0.018 mm^−1^ (CI 95%, 0.190–0.200 mm^−1^) EAC_830_ = 0.178 ± 0.018 mm^−1^ (CI 95%, 0.173–0.183 mm^−1^). In comparison, they obtained a minimum EAC in parietal regions: EAC_690_ = 0.173 ± 0.026 mm^−1^ (CI 95%, 0.166–0.180 mm^−1^), EAC_830_ = 0.160 ± 0.026 mm^−1^ (CI 95%, 0.153–0.167 mm^−1^). These results suggested a maximum regional variability between 20–25% at a 95% confidence interval, which is a smaller variability compared to the one we obtained. This discrepancy may depend on the different spatial sensitivities of multi-distance CW recordings with respect to TD systems, or to the lower spatial sampling used by Giacalone and colleagues (a total of 12 points across the scalp), which could decrease the strength of the estimated regional effect on EAC.

These spatial differences may have an important influence in determining the penetration of fNIRS measurement in different parts of the head, with greater penetration in lateral and frontal regions than in dorsal and occipital areas. Previous studies had proposed that distance between the brain surface and the scalp may be an important factor in the sensitivity of fNIRS measurement to cortical phenomena (e.g., [62,67]), and that therefore sensitivity may vary over the head and perhaps across individuals. However, unlike the current study, these studies were based on simulated models rather than on recorded data. Further, the data reported here indicate that regions with the highest EAC values largely correspond to areas where large veins are present in the tissue. These results are consistent with our previously published paper [20] where we hypothesized this difference to be due to the presence of large veins in these regions [68].

Several features of the data strongly indicate that EAC values provide information related to anatomical properties of the head, and in particular of the brain and surrounding tissue. First, the average EAC maps across wavelengths reported in this study were very consistent with each other. This supports the idea that local variations in EAC value reflect anatomical features, rather than random variations. Further, the EAC’s SEs (Figure 2b) were relatively small and were generally larger (when measured as a percent of the mean value across individuals) in areas where the spatial gradients of the average EAC maps were large. While these data supported the overall consistency of the average EAC maps, they also hinted at the existence of some variability in the exact locations of the EAC maxima across individuals. A plausible interpretation is that this may reflect individual differences in the location of large blood vessels (and in particular veins) in the head, which, because of the large concentration of hemoglobin, produce local peaks in light absorption (and therefore in EAC). Overall, these data suggest that information about the specific head anatomy of each individual may be very useful when modeling how light propagates in the brain (see [67] for a similar claim), but also that such information should include a way of estimating the effect of large blood vessels (such as veins and arteries) in each individual. T1w sMRI images typically do not include veins and arteries, which instead require arteriograms and venograms. Alternatively, the EAC-estimation approach presented here may provide a tool for generating maps of optical properties from each individual’s data (albeit at lower resolution than sMRI), without the need for additional recording and without the need for building realistic head models.

In addition to the local features identified through mapping (mostly related to the location of blood vessels), the current study also indicates that whole-head average EAC estimates for each individual and wavelength may provide data about the average thickness of the CSF-filled sub-arachnoid space surrounding the brain, and as consequence, about brain (and especially cortical) atrophy. Note that the average data indicated a correlation between the EAC values observed at the two wavelengths. This correlation can be considered to be a minimum estimate of the reliability of the average EAC values (as the two estimates were obtained on independent sets of data taken from each individual). That said, it is likely to under-estimate reliability because some of the inter-wavelength variability is probably due to true variance (e.g., variance due to differences in oxygenation across individuals) rather than error variance. This reliability is very high, exceeding *r* = 0.85.

Notwithstanding the high correlation between the EAC values at the two wavelengths, EAC was consistently larger at 690 nm than at 830 nm. This indicates that light at longer wavelengths has a deeper penetration in the head than light at shorter wavelengths, corresponding to the idea that the average photon path-length factor is different at these two wavelengths (see Uludag et al. [69] for an evaluation of the impact of this difference on applications of the Beer-Lambert law for the computation of oxy- and deoxy-hemoglobin concentrations).

Interestingly, the average EAC was not correlated with sex, nor did we find any effect of sex on the age–EAC association. However, EAC was strongly and negatively correlated with age (Figure 4a,b) and positively correlated with cortical thickness (Figure 5a-d), and to a lesser degree with cortical volume. A negative association between age and absolute optical properties was also found by Giacalone and colleagues [66] using TD technology. By performing a split-half analysis as a function of age, they found a decrease in EAC (derived from the reported absorption and reduced scattering coefficient) in older (>55 years of age) subjects: EAC_690_ = 0.184 ± 0.0187 (CI 95%, 0.179–0.189 mm^−1^), EAC_830_ = 0.166 ± 0.013 (CI 95%, 0.163–0.170 mm^−1^) compared to younger (<55 years of age) subjects: EAC_690_ = 0.203 ± 0.024 (CI 95%, 0.193–0.213 mm^−1^), EAC_830_ = 0.182 ± 0.016 (CI 95%, 0.176–0.189 mm^−1^). These results are comparable with the age-related effect on EAC reported in this paper. In fact, if we perform a split-half analysis as a function of age instead of a correlation analysis, we obtain comparable results, with average EAC values of EAC_690_ = 0.175 ± 0.044 mm^−1^ (CI 95%, 0.163–0.188 mm^−1^), EAC_830_ = 0.150 ± 0.034 mm^−1^ (CI 95%, 0.140–0.159 mm^−1^) in the older group and EAC_690_ = 0.21 ± 0.030 mm^−1^ (CI 95%, 0.201–0.219 mm^−1^), EAC_830_ = 0.185 ± 0.030 mm^−1^ (CI 95%, 0.176–0.194 mm^−1^) in the younger group.

To better understand the relationship of EAC with age and cortical thickness, we further computed separate EAC estimates based on channels with different source–detector distances. Results suggested that the correlations between EAC, age, and cortical thickness were driven by phenomena occurring below the most superficial head layers. In fact, the EACs computed using channels with a short source–detector distance (15–25 mm), and mostly investigating the skin and skull but with little penetration into the brain [62], did not show any statistically significant correlation with either age or cortical thickness. These correlations, however, were evident when longer source–detector distances, with greater sensitivity to the brain were considered [62]. This suggests that EAC variations across ages most likely reflect variations in brain anatomy or physiology rather than variations in skin or skull. The low correlation found at the longest inter-optode distances (45–55 mm) could be due to a decrease in SNR at such distances. Alternatively, it could reflect a real phenomenon: aging influences intermediate layers (such as the relative thickness of the CSF and cortical layers—consistently with the correlation between EAC and cortical thickness) rather than deeper layers (note, however, that a shift of the cortical layers toward deeper regions due to shrinking of the underlying white matter could also account for some of these effects).

Note that the aim of this analysis was only to determine whether the age effect on EAC was due to phenomena occurring in the most superficial or in deeper layers of the head by exploiting the monotonic relation between source–detector distance and depth sensitivity in back-reflection diffuse optical imaging. No inference on the depth of the effect was provided. The EAC estimation algorithm, since it requires multiple sources and detectors, is poorly suited for high-resolution 3D imaging (diffuse optical tomography, DOT). Such a goal would require specific development of image reconstruction algorithms, and, presumably, an iterative approach. In fact, in standard DOT, each channel is used as a standalone measurement, and the image is reconstructed by exploiting the different sensitivities of each channel to phenomena at different depths, which themselves would be influenced by EAC. The methodology is complex and would definitely require validation. Therefore, we preferred to only provide here a topographic mapping of EAC.

A series of mediation analyses indicated that the age–EAC association becomes non-significant when the impact of age-related changes on cortical thickness is partialed out. This is consistent with the idea that the age-related effect on EAC is, for the most part, related to brain atrophy, either directly (perhaps because a thinner cortex is less of an obstacle to light transmission than a thicker cortex) or, more likely, indirectly (reflecting the fact that lost cortical or sub-cortical tissue is replaced by the more transparent CSF). In any case, this finding supports the idea that, through estimation of the EAC, diffuse optical imaging data can provide information about the level of brain atrophy in an individual, in addition to providing more accurate path lengths for spectroscopic applications and for the 3D reconstruction of fNIRS data.

There are also hints in the data that the average EAC provides not only anatomical but also physiological information. In fact, application of the TOI method proposed by Suzuki et al. [26] suggests that differences between the average EAC values obtained at 690 nm and at 830 nm may provide an estimate of the oxygen saturation of brain tissue. The estimates obtained with this approach are similar to previously reported estimates obtained with TD methods [65]. The fact that these EAC-based estimates of tissue oxygenation are significantly and negatively correlated with age, heart rate variability and cardiorespiratory fitness corroborates this interpretation.

A possible problem with this interpretation, however, is that the absolute EAC value might be somewhat correlated with the TOI estimate, since they are both extracted from the same data. To understand whether in fact they correspond to separate (orthogonal) sources of variance we performed a PCA (Eigen-decomposition) of the EAC values obtained at 690 and 830 nm for each subject. The results of this PCA indicated that the variance in EAC values observed at the two wavelengths for each subject can be considered as reflecting two linearly independent (orthogonal) latent variables. The first latent variable (accounting for most of the variance) is related to structural changes that affect both wavelengths similarly (i.e., with very similar component loadings). The strong correlations of this latent variable with age and cortical thickness indicate that it is really reflecting anatomical features (brain shrinking and/or enlargement of the CSF space, which often occur with age). In contrast, the second latent variable is strongly dependent on wavelength (Figure 7b), with opposite sign loadings for the two wavelengths. This second component is very strongly correlated with TOI (∣*r*∣ > 0.97). The wavelength dependence of this second PC is obviously driven by the differential sensitivity of the two wavelengths to oxy- and deoxy-hemoglobin (given by the fact that they are at the opposite sides of the hemoglobin’s isobestic point). Thus, this second PC could be useful for providing an absolute estimate of the tissue oxygenation level in the brain, both averaged across the entire brain and separately for each region. Further, these data demonstrate that the anatomical (brain shrinking) and physiological (tissue oxygenation) information provided by EAC are independent of each other.

The hypothesis that the first EAC eigen-solution (or component—EAC1) is related to cortical thickness, and therefore to cortical atrophy, is further supported by its correlation with a large number of anatomical, physiological, and cognitive variables. Some of these correlations remained significant even when the effect of age was partialed out, although the strong collinearity between age and EAC1 generally reduces the size of the correlations. In general, the patterns of correlations between EAC1 and all these variables are very similar to that of cortical thickness (*r* = 0.943), and this similarity in pattern is visible even when age is partialed out from all the correlations (*r* = 0.739), indicating that it cannot be attributed to the common influence of age on all the variables investigated.

The hypothesis that the second EAC eigen-solution or component (EAC2) is related to tissue oxygenation is less strongly supported. However, the pattern of correlations with the other variables is very similar to that of CRF and heart rate variability (respectively, *r* = −0.804 and *r* = −0.810). Further, the similarities of these patterns are largely maintained when age is partialed out from all the correlations (with *r* = −0.662 for the correspondence between the EAC2 and CRF patterns, and *r* = −0.632 for the correspondence between the EAC2 and heart rate variability patterns). These results provide indirect validation for the claim that EAC2 is related to the trophic and vascular state of tissue.

Finally, analyses of the correlations between local measures of EAC1 and demographic, physiological, and cognitive variables reveal interesting patterns. Several of these maps indicate that the strongest correlations could be observed when the EAC1 is estimated from optical probes located over the frontal and parietal regions. This pattern is especially evident for the O-Span measure, an index of working memory function. This is consistent with the idea that frontal and parietal cortex play an important role in working memory function, and that losses in cortical thickness in these regions may be associated with declines in working memory abilities [70]. Note also that cardiorespiratory fitness presents a similar pattern of correlations, which is also consistent with neuroanatomical studies indicating that the effects of CRF are particularly evident in frontoparietal cortex [22]. By-and-large, these data suggest that the average EAC1 can be effectively measured not only across the whole head, but separately for different regions, and that these measures provide information about the level of atrophy in different regions of the brain.

In future research it would be interesting to further investigate the spectral dependence of EAC through spectroscopic studies involving additional NIR wavelengths. Although here we computed EAC values in healthy adults, an important application of EAC mapping would be to investigate people with pathological conditions that affect both oxygenation and anatomy (e.g., superficial hemorrhages, strokes, etc.), or where subtle, regional and perhaps temporary physiological [71] or pathological alterations of oxygenation (such as concussion [72]) are suspected. In fact, in this paper we only considered the generation of static EAC maps (i.e., the average maps across the recording period) to maximize SNR. In future work we will investigate the time-dependence of EAC, where EAC images will be obtained at high sampling rates (up to few Hz). This may provide quantitative functional imaging data, describing, for instance, the percent change in oxygenation in response to stimuli or during a particular task. Coupled with pulse-DOT measures of arterial status [15], the EAC and TOI indices could also be used to monitor brain oxygenation in preterm infants within the incubator [16] together with the monitoring of other standard physiological parameters.

## 5. Conclusions

In conclusion, this paper presents the first systematic mapping of absolute optical properties (EAC) over the whole head employing CW technology in a large healthy sample of adults over a broad age range, showing that these data are consistent with results obtained using smaller TD systems. The mapping procedures reported here may be useful to improving the accuracy of oxy- and deoxy-hemoglobin estimation and 3D reconstructions of fNIRS data. Most importantly, given the dependence of the EAC on regional anatomical (cortical thickness) and physiological (tissue oxygenation) factors, calculating the EAC may also enable the retrieval of such information from fNIRS recordings directly, albeit at low spatial resolution. In particular, average EAC levels across areas of the scalp may provide indications of brain atrophy, a parameter of great clinical importance, as well as, perhaps, indices of tissue oxygenation, another parameter that might have potential clinical value.

## Figures and Tables

**Table 1.** Correlations between the first and second EAC eigen-solutions (EAC1 and EAC2), age, and other demographics, vascular, anatomical, and cognitive variables. The table also includes correlations of these variables with EAC1 and EAC2 with age partialed out. Perf.-Verb. Score is the difference between the performance and verbal score; N = 47; **Bold** = *p* < 0.05; *Italics* = *p* <0.10.

Variable	EAC1	EAC2	Age	EAC1.Age	EAC2.Age
Age	**−0.496**	*0.275*			
CRF	**0.332**	−0.247	**−0.741**	−0.062	−0.067
Sex (1 = M, 2 = F)	0.230	0.183	0.034	*0.284*	0.180
Education (Yrs)	−0.155	0.027	**0.492**	0.119	−0.129
Heart Rate (bpm)	−0.042	0.068	−0.144	−0.132	0.113
Heart Rate Var. (ms)	0.172	**−0.334**	**−0.635**	−0.213	−0.215
PReFx (overall)	**0.423**	−0.049	**−0.409**	*0.278*	0.072
PReFx (L Hem)	**0.438**	−0.010	**−0.392**	**0.305**	0.110
PReFx (R Hem)	**0.388**	−0.085	**−0.407**	0.235	0.030
Cortical Thickness (mm)	**0.575**	−0.034	**−0.658**	**0.380**	0.204
WMSA (log voxels)	**−0.534**	0.014	**0.552**	**−0.359**	−0.172
Performance Score	**0.457**	−0.150	**−0.471**	*0.291*	−0.024
Verbal Score	−0.194	0.006	**0.430**	0.025	−0.129
Perf.-Verb. Score	**0.433**	−0.099	**−0.617**	0.185	0.093
mMMS	−0.161	−0.058	0.168	−0.091	−0.110
KBIT	−0.186	0.112	0.172	−0.118	0.069
Raven’s Matrices	**0.293**	−0.102	**−0.387**	0.126	0.005
Shipley Vocabulary	−0.186	0.245	**0.457**	0.052	0.139
Forward Digit Span	−0.078	0.011	**0.351**	0.117	−0.095
Backward Digit Span	−0.192	−0.016	0.270	−0.069	−0.098
Verbal Fluency	−0.069	**−0.297**	0.127	−0.007	**−0.348**
O-Span	**0.417**	−0.089	**−0.454**	0.248	0.042
Trail A (sec.)	*−0.260*	0.226	*0.271*	−0.150	0.164
Trial B (sec.)	**−0.364**	0.165	**0.290**	*−0.265*	0.092
Trail B-A (sec.)	**−0.293**	0.085	0.206	−0.225	0.031
